# Bone missile injury with an intact host femur after high energy road traffic trauma: a case report

**DOI:** 10.1093/jscr/rjag903

**Published:** 2026-10-10

**Authors:** Ezekiel Baguma, Mark Jjuuko, Paul Kabazzi, Daniel Ezati, Phillip Mulepo, Peter Muwanguzi

**Affiliations:** Department of Orthopaedic Surgery, Makerere University College of Health Sciences, Kampala, Uganda; Department of Orthopaedic Surgery, Masaka Regional Referral Hospital, Masaka, Uganda; Department of Orthopaedic Surgery, Masaka Regional Referral Hospital, Masaka, Uganda; Department of Orthopaedic Surgery, Makerere University College of Health Sciences, Kampala, Uganda; Department of Orthopaedic Surgery, Makerere University College of Health Sciences, Kampala, Uganda; Department of Orthopaedic Surgery, Makerere University College of Health Sciences, Kampala, Uganda

**Keywords:** bone missile, bony foreign body, post-exposure prophylaxis, resource-limited setting

## Abstract

A 28 year old woman presented with local wound infection two weeks after sustaining an anterior thigh laceration in a high energy motorbike crash that killed the co-riding motorcyclist. X-ray revealed a non-identifiable radio-opaque fragment and computed tomography identified a bony foreign body deep in the thigh adjacent to the femoral vessels, with the host femur intact. A 6 x 3 cm fragment of cortical bone was removed at surgery. Histopathology and genetic studies to confirm its origin were not feasible in this resource-limited setting, but an intact host femur suggested it originated from the deceased rider. The wound healed by secondary intention, HIV and hepatitis serology were negative at 6 weeks. This case highlights the unusual occurrence of a bone missile and the importance of early advanced imaging and prompt post-exposure prophylaxis following high-energy, multi-victim trauma.

## Introduction

Foreign body implantation in soft tissue following trauma is a known entity and the transfer of bone fragments between two distinct trauma patients, known as ‘bone missile’ is very rare [1, 2]. Biological fragments can be thrown from a high energy road traffic crash with enough force to penetrate a bystander or co-victim, posing a diagnostic challenge since the foreign material might not be immediately visible on the initial X-ray or other plain film imaging and is a potential risk for blood-borne pathogen transmission. A large piece of bone from the cortex of a fractured femur from a road traffic accident victim, which was thought to have come from the other party who had died at the scene, is reported and the lessons to be learned from the diagnosis and infection control are discussed.

## Case report

Two weeks after being a passenger on a motorbike which crashed in a head-on accident, killing the rider, a 28-year-old woman was referred to an orthopaedic outreach camp at a regional referral hospital in Masaka, Uganda for treatment of an isolated deep anterior thigh laceration. She was treated with tetanus toxoid, wound debridement, and primary closure at the referring hospital and the radiographs revealed no evidence of a fracture to the femur and no foreign body was identified. On examination at review the wound was infected locally with erythema and purulent discharge around it (Fig. 1). A plain radiograph showed that the fragment was a dense radio-opaque object that was superimposed on the soft tissues in the proximomedial thigh next to the proximal femur (Fig. 2). High-resolution computed tomography (HRCT) with 3D reconstruction revealed a large bony fragment in the deep area of the femoral triangle in close proximity to the femoral neurovascular bundle, while there was a complete femur (Fig. 3). Wound exploration and debridement under spinal anaesthesia did produce a hard irregular 6 × 3 cm piece of cortical bone, adjacent to the femoral vessels (Fig. 4). Due to the resource limitations at this site, a diagnosis could not be established by genetic or histopathological analysis and an intact host femur was the only basis for identification, while culture and sensitivity testing of a wound swab was also not possible and antibiotic therapy was therefore empirical, with use of intravenous broad-spectrum agents. It was decided to treat the wound open, with healing by secondary intention because of the soft tissue deficiency. The biological nature of the fragment was not known until two weeks after the injury, precluding the possibility of starting HIV post-exposure prophylaxis within the recommended 72 hours; HIV status of the deceased rider was unknown. HIV antibody/antigen test at presentation was negative and repeat HIV, hepatitis B and hepatitis C serology at 6 weeks was negative.

**Figure 1:**
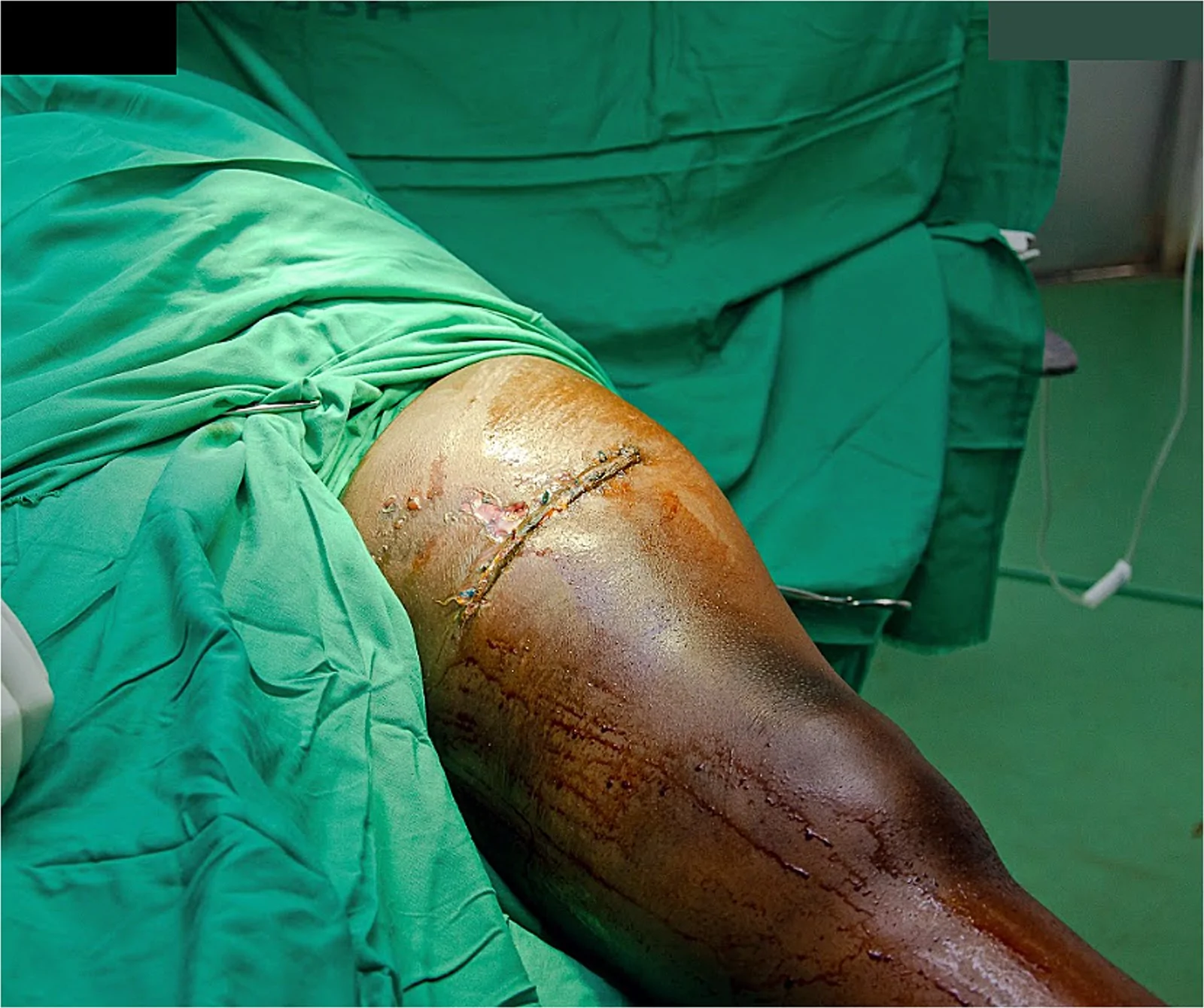
Clinical photo from presentation of localized wound infection from sutured laceration on the anterior left thigh.

**Figure 2:**
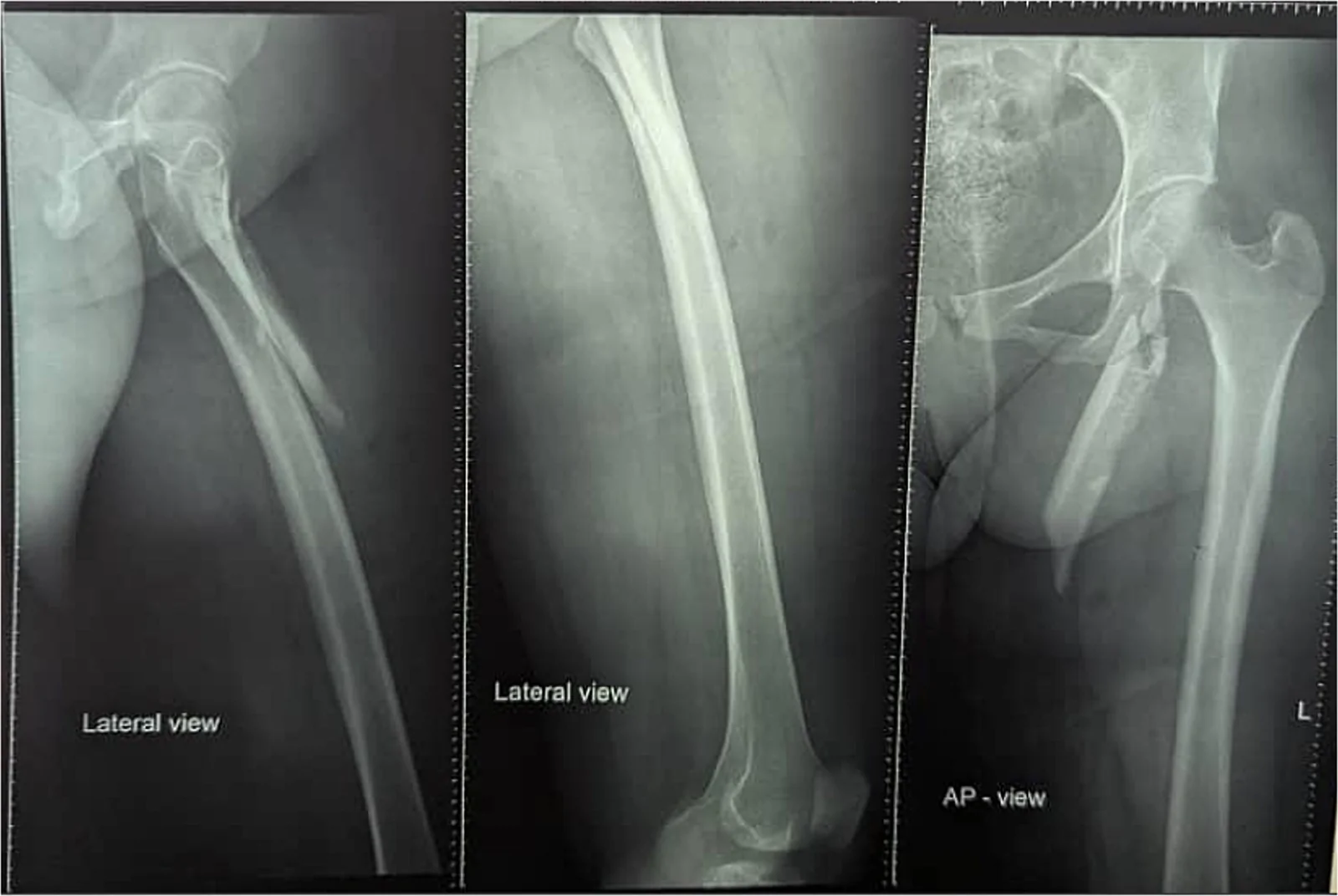
Initial plain radiograph showing a dense, radio-opaque fragment over the proximomedial thigh soft tissues in which there is no evidence of a fracture of the femur.

**Figure 3:**
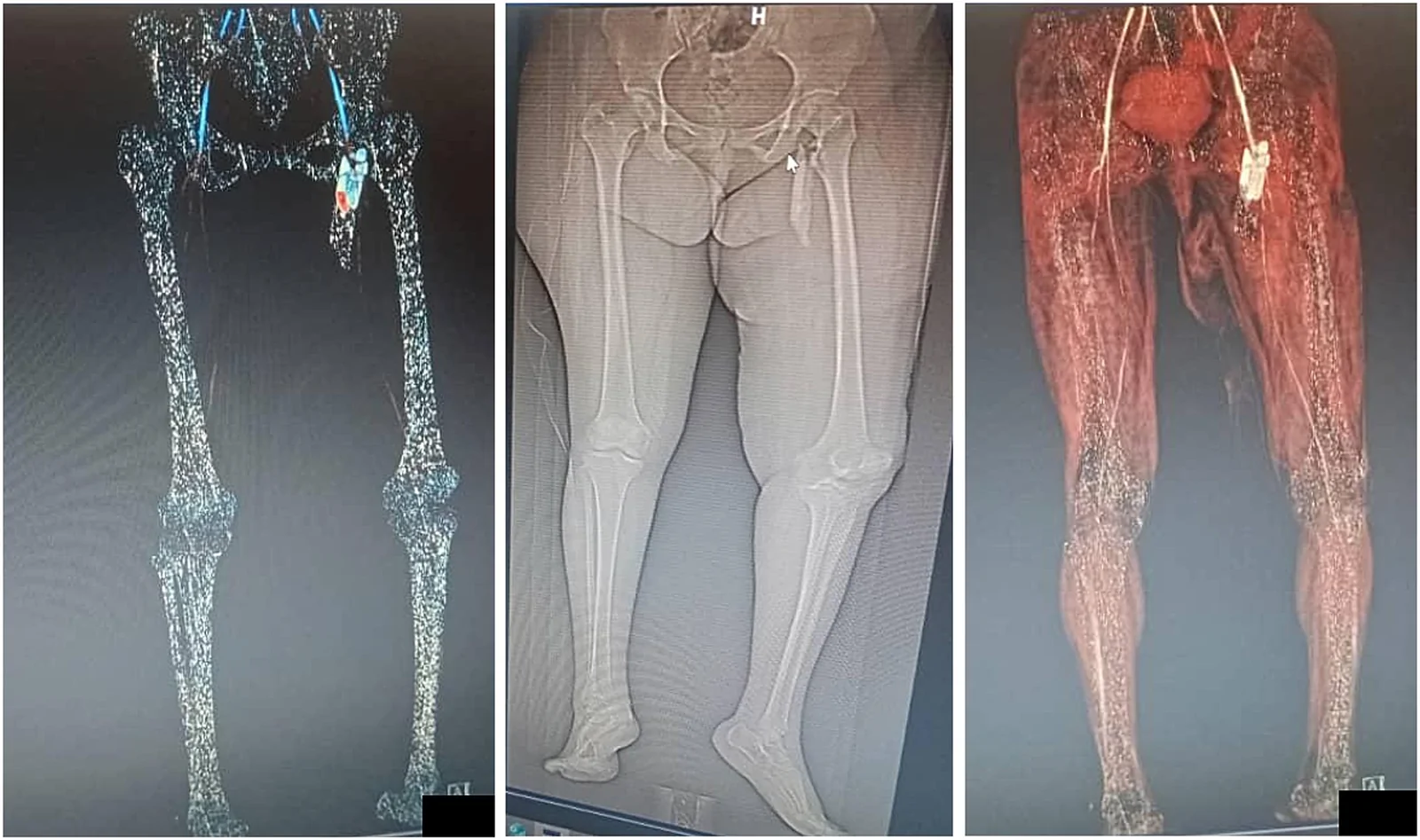
HRCT 3D images, showing a bony foreign body very close to the femoral vessels and confirming an intact left femur.

**Figure 4:**
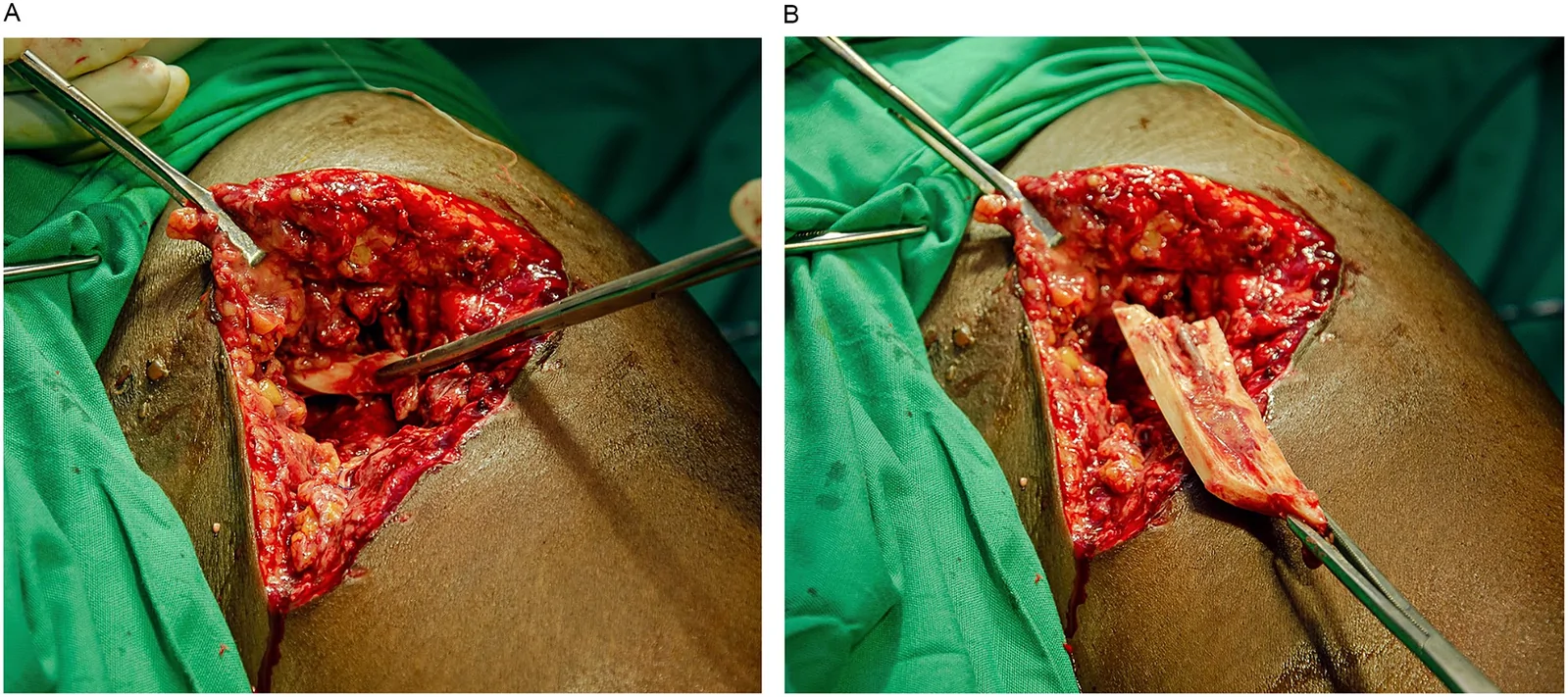
Intraoperative photographs during wound exploration (A) and the retrieved (B) 6 × 3 cm cortical bone fragment.

The patient had no known comorbidities and no other contributory past medical history. A full blood count on admission showed leukocytosis (white cell count 17.8 × 10^2^/μl) with neutrophil predominance (87.3%) and mild anaemia (haemoglobin 8.9 g/dl, mean corpuscular volume 81.4 fl), findings consistent with the local wound infection. Renal function tests and inflammatory markers (C-reactive protein/ erythrocyte sedimentation rate) were not obtained in this resource-limited outreach setting. At the last clinic review, three months after injury, the wound had healed completely by secondary intention and the patient had returned to her pre-injury level of activity, with no residual pain, weakness, or neurovascular deficit in the limb.

## Discussion

This case is an unusual example of deeply embedded large foreign body (presumably of human origin) after high energy trauma in the deep soft tissues of another man. There is sparse literature on bone missile injuries and most of the case reported in literature describes smaller missiles, our case is remarkable with the size of the missile and its close relationship with the major neurovascular bundle, similar to the described case by Kaushik *et al.* and Alsafty [1, 2]. In this case, the foreign body was not noticed by the primary doctor until it was found on the initial radiographs, demonstrating how foreign body can remain unnoticed on primary radiographs and the usefulness of HRCT and three dimensional reconstruction of images for localization and surgical planning. A critical learning moment was the missed window of opportunity for post-exposure prophylaxis for HIV in the 72 hours after exposure: in the multi-victim road traffic collisions where there has been a fatality, clinicians should have a high index of suspicion for biological foreign body implantation, and should promptly begin blood-borne pathogen risk assessment and post-exposure prophylaxis without waiting for definitive identification of the foreign body, even in sub-Saharan Africa where HIV prevalence is high [3, 4]. This was necessary due to the high level of contamination possible with the presence of biological foreign bodies; careful dissection eliminated unnecessary vascular injury. Limitations of genetic and histopathological testing of the fragment were not able to confirm the human origin; confirmatory HIV nucleic acid testing was not available; these limitations reflect the resource-limited, outreach-based setting in which the patient was managed, and led to reassurance through serial serology. This case highlights the importance of high index of suspicion for retained foreign bodies in high-energy trauma, the use of CT imaging in high-energy trauma when there is a possibility of a retained foreign body, avoiding primary closure in contaminated high-energy wounds and early consideration of HIV post-exposure prophylaxis when there is a risk of biological cross-contamination.

Direct comparison with the two previously published bone missile cases is limited by the level of clinical detail available in their published reports. Both Alsafty and Kaushik *et al.* describe, as in the present case, a large bony fragment transferred from a co-victim of a high-energy road traffic collision and lodged deep in the soft tissues of the surviving patient, in close proximity to major neurovascular structures, with the host bone itself intact. These findings are closely parallel to our patients presentation [1, 2]. However, granular comparative data on patient demographics, definitive confirmation of fragment provenance, and long-term functional outcome are not consistently reported in these publications. The present case adds to this limited literature by documenting the diagnostic pathway from an initially unremarkable radiograph to HRCT with 3D reconstruction for precise localization and surgical planning; the practical challenges of confirming biological origin and infective risk without access to histopathological, genetic, or microbiological testing in a resource-limited outreach setting; and a defined clinical and serological follow-up (to 6 weeks for blood-borne virus screening and three months for functional recovery), with complete wound healing by secondary intention and full return to pre-injury activity. These management and follow-up details, only partially addressed in the existing reports, may help standardize the diagnostic and infection-control approach to bone missile injuries encountered in similar high-energy, multi-victim trauma settings.
